# Optimization of Smooth Trajectories for Two-Wheel Differential Robots Under Kinematic Constraints Using Clothoid Curves

**DOI:** 10.3390/s25103143

**Published:** 2025-05-15

**Authors:** Wei Zeng, Tifan Xiong, Chao Wang

**Affiliations:** School of Mechanical Science and Engineering, Huazhong University of Science and Technology, Wuhan 430074, China; weizeng_m@hust.edu.cn (W.Z.); xiongtf@hust.edu.cn (T.X.)

**Keywords:** differential drive, trajectory planning, clothoid curve, optimization, kinematic constraints

## Abstract

Navigation is a fundamental technology for mobile robots. However, many trajectory planning methods suffer from curvature discontinuities, leading to instability during robot operation. To address this challenge, this paper proposes a navigation scheme that adheres to the kinematic constraints of a two-wheeled differential-drive robot. An improved and efficient RRT algorithm is employed for global navigation, while an adaptive clothoid curve is utilized for local trajectory smoothing. Simulation results demonstrate that the proposed method effectively eliminates curvature discontinuities and enhances operational efficiency.

## 1. Introduction

Mobile robots have become an integral part of modern applications, including cleaning, industrial automation, and warehousing [1]. They are also well suited for replacing humans in tasks that are monotonous or hazardous or require operation in challenging environments. To achieve autonomy, a mobile robot must possess the ability to perceive its surroundings and plan a collision-free trajectory from the starting position to a designated target [2]. Path planning plays a crucial role in this process and is generally categorized into global and local path planning, depending on the availability of complete environmental information [3].

Global path planning algorithms encompass well-established techniques such as Rapidly-Exploring Random Tree (RRT), the ant colony algorithm [4], the bee colony algorithm [5], particle swarm optimization [6], Dijkstra’s algorithm [7], and the A* algorithm [8]. In contrast, local path planning methods primarily include the Dynamic Window Approach (DWA) [8], the Timed Elastic Band (TEB) [9], and the artificial potential field (APF) method [10]. The paths generated by these algorithms are typically composed of discrete straight-line segments and abrupt turns [11], which are suboptimal for direct execution due to inefficiencies in motion smoothness and control feasibility.

Trajectory planning refines a reference path to produce a dynamically feasible and executable trajectory for a mobile robot. This process must account for kinematic constraints to ensure smooth motion, stability, and feasibility during execution. While traditional path planning techniques provide collision-free paths, they often overlook the robot’s dynamic characteristics, leading to sudden turns or abrupt acceleration changes that degrade navigation performance. Consequently, optimizing the trajectory to conform to the robot’s kinematic constraints while enhancing smoothness has become a critical research focus in mobile robot navigation.

In practical applications, mobile robots frequently encounter complex system challenges such as nonlinear dynamics, input constraints, and actuator faults. Existing research predominantly centers on maintaining system stability and ensuring reliable task execution. For instance, fault-tolerant control strategies that integrate control barrier functions (CBFs) with reinforcement learning have demonstrated the ability to uphold safety under unknown disturbances [12]. Similarly, robust approaches grounded in differential game theory have proven effective for modeling intricate tasks with state constraints [13]. Additionally, lightweight neural network architectures designed for edge deployment have gained traction in visual defect detection tasks within resource-limited environments [14,15]. While these works do not directly address trajectory planning, they collectively underscore the critical importance of stability and physical executability during the task execution phase.

This study primarily focuses on generating smooth trajectories with continuous curvature. A widely adopted approach in this domain is path smoothing, which refines an initially coarse reference path to ensure curvature continuity and meet smoothness requirements. Among the various path smoothing techniques, curve-based methods are frequently employed to interpolate sparse waypoints and generate continuous and feasible trajectories.

One popular category within this approach is spline interpolation, such as Catmull–Rom splines, which have attracted significant attention due to their ability to construct smooth and continuous curves using Lagrange interpolation and B-spline basis functions [16]. In particular, cubic Catmull–Rom splines have been used to generate trajectory segments at intersections while preserving continuity at junction points [17]. However, a key limitation of spline-based methods lies in their restricted local control, which makes it difficult to finely adjust curve shapes to meet specific trajectory constraints.

Beyond spline-based approaches, Dubins curves and Reeds–Shepp curves provide alternative solutions for computing the shortest path between two configurations while respecting vehicle kinematic constraints [18,19]. Despite their effectiveness in satisfying motion constraints, these methods often require additional collision-checking steps, as the resulting paths may deviate substantially from the original reference trajectory.

Bézier curves offer another flexible trajectory generation strategy by defining paths through a series of control points [20]. They have been extensively studied for path smoothing [21,22,23,24], owing to their capability to produce smooth trajectories. However, controlling the curvature of Bézier curves remains challenging, particularly when compared to clothoid curves, which feature a linear change in curvature and are thus well suited for generating gradual and smooth transitions during motion planning.

Originally proposed by the French mathematician Euler, the clothoid curve has been widely applied in autonomous vehicle applications, particularly in automated parking scenarios [25,26]. It has also been adopted in many studies for generating smooth and feasible trajectories [27]. Nevertheless, existing implementations often fail to consider the acceleration constraints specific to two-wheeled differential-drive robots, limiting their ability to fully satisfy the practical motion requirements of such systems.

Beyond path smoothness, practical trajectory planning must also consider dynamic constraints, such as velocity, acceleration, and jerk, to ensure smooth motion while maintaining system feasibility. Additionally, minimizing the trajectory execution time is crucial for improving the responsiveness and overall efficiency of the system. However, enforcing velocity, acceleration, and jerk constraints inherently limits the extent to which the trajectory duration can be minimized. To address this issue, time-optimal trajectory planning techniques have been developed to strike a balance between execution time minimization and adherence to motion feasibility constraints.

As a result, ongoing research is increasingly focused on developing advanced trajectory planning algorithms that can adapt to various dynamic scenarios while optimizing overall vehicle performance [28]. Among the local trajectory planning methods, several approaches have been extensively studied, including techniques based on lateral acceleration [29], network search algorithms [30], sampling-based motion planning [31,32], and artificial potential field (APF) methods [33,34]. To enhance solution space flexibility, Ref. [35] proposed an optimization framework that utilizes trapezoidal prism-shaped corridors, offering significant improvements over traditional cuboidal corridor-based methods. Similarly, Ref. [36] introduced a spatial traversability mapping approach tailored for urban driving, though its high computational complexity remains a limitation for large-scale trajectory generation.

Further advancements in trajectory generation have introduced second-order Bézier curve-based techniques [37], which, although effective in specific applications, face limitations in handling complex and dynamic environments. To overcome the constraints associated with predefined reference trajectories, Ref. [38] proposed a trajectory generation framework that accounts for system constraints and produces multiple candidate paths. However, the performance of such methods remains sensitive to factors such as model accuracy, computational overhead, and real-time adaptability. In parallel, Ref. [39] developed an improved artificial potential field (APF)-based trajectory planning method that integrates adaptive distance modulation, dynamic road repulsion, and velocity-constrained planning. While these enhancements improve trajectory feasibility, APF-based methods are inherently susceptible to local minima, which may result in suboptimal paths or navigation deadlocks. The TubeSTRRT* algorithm notably improves the physical executability of trajectories by incorporating a trajectory optimization mechanism [40]. Moreover, the integration of Bézier curves with time parametric frameworks allows for compliance with speed constraints while ensuring smooth transitions in control commands [41]. Additional approaches such as eliminating pseudo-acceleration discontinuities via quadratic polynomial interpolation [42] and enhancing trajectory continuity using spline interpolation strategies, as demonstrated in the NC-BRRT algorithm [43], provide valuable perspectives on optimizing trajectory smoothness.

**Figure 1:**
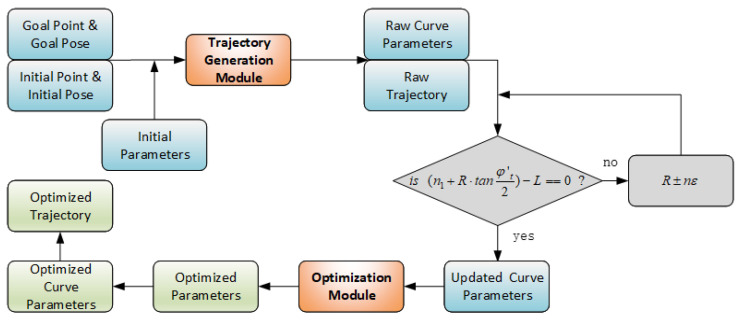
Flowchart of the proposed method.

To address these challenges, this paper proposes an optimized trajectory generation method based on clothoid curves for two-wheeled differential-drive mobile robots. The proposed approach integrates both trajectory and velocity planning, aiming to minimize the execution time while ensuring smooth transitions. Ultimately, it generates an optimal and dynamically feasible trajectory tailored to two-wheeled differential-drive systems. A flowchart illustrating the proposed method is presented in Figure 1. The remainder of this paper is organized as follows: Section 2 presents the fundamental modeling of the clothoid curve. Section 3 introduces the proposed trajectory generation and parameter optimization modules. Section 4 provides the experimental results, and Section 5 concludes the paper.

## 2. Preliminaries

### 2.1. Clothoid Curve

A clothoid curve is a type of relaxation curve. Its most significant feature is that the curvature changes linearly with the path length. The mathematical form is

(1) $$\frac{dk}{dl}=\pm a$$

In this equation, *k* represents the curvature of the clothoid curve, and *l* is the total arc length of the clothoid curve from curvature 0 to *k*. The symbol ± indicates whether the curvature increases or decreases along the curve. The *a* parameter is a constant that determines the rate of curvature change—the larger the *a* parameter, the faster the curvature varies.

### 2.2. Curve Model Building

For a point (x,y,θ) on any curve, the following three differential relations hold, where *x* and *y* denote the coordinates of the point, θ represents the tangent angle, and *k* is the curvature of the curve:

(2) dθ=kdl

(3) dx=dl·cosθ

(4) dy=dl·sinθ

If the curve is parameterized by the arc length *l* and curvature k(l), the pose $\left(x_{h},y_{h},\theta_{h}\right)$ at arc length *L* can be calculated as follows:

(5) $$\theta_{h}=\theta_{0}+\int_{0}^{L}k\left(l\right)\;dl$$

(6) $$x_{h}=x_{0}+\int_{0}^{L}\cos\left(\theta \left(l\right)\right)\;dl$$

(7) $$y_{h}=y_{0}+\int_{0}^{L}\sin\left(\theta \left(l\right)\right)\;dl$$

In this case, θ(l) can be decomposed as

(8) θ(l)=β(l)+γ

where β(l) is a function of *l*, and γ is a constant independent of *l*. Hereafter, β(l) is abbreviated as β for notational simplicity, and the above equations can be rewritten as follows:

(9) $$x_{h}=x_{0}+\cos\gamma \cdot \int_{0}^{L}\cos\beta \;dl-\sin\gamma \cdot \int_{0}^{L}\sin\beta \;dl$$

(10) $$y_{h}=y_{0}+\sin\gamma \cdot \int_{0}^{L}\cos\beta \;dl+\cos\gamma \cdot \int_{0}^{L}\sin\beta \;dl$$

**Figure 2:**
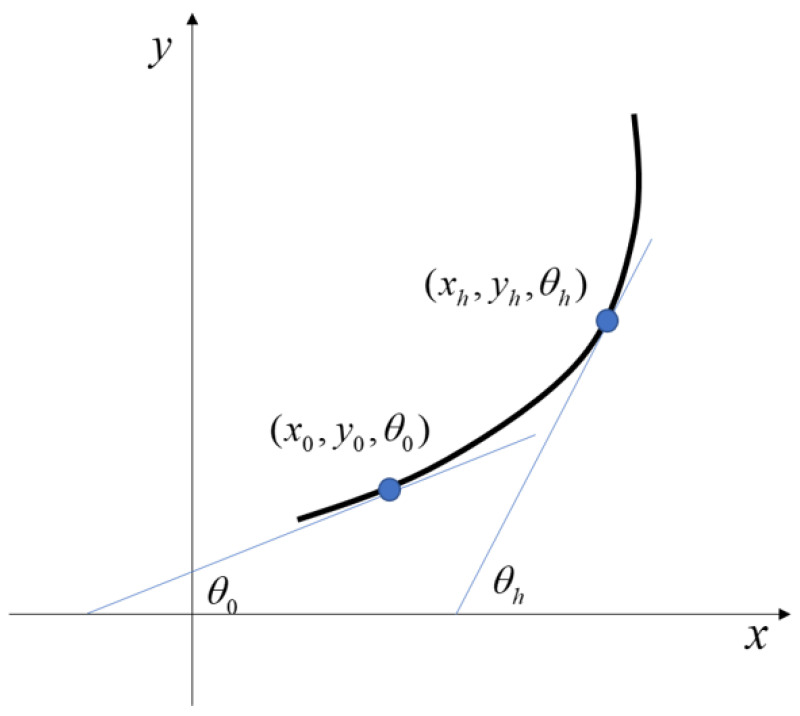
Turning model.

Here, $\left(x_{0},y_{0},\theta_{0}\right)$ represent the coordinates and tangent angle at the starting point of the curve, as illustrated in Figure 2.

According to the Taylor series expansions of the trigonometric functions, the following relationships hold:

(11) $$\cos\beta =\sum_{n=0}^{\infty }\frac{{\left(-1\right)}^{n}\beta^{2n}}{(2n)!}$$

(12) $$\sin\beta =\sum_{n=0}^{\infty }\frac{{\left(-1\right)}^{n}\beta^{2n+1}}{(2n+1)!}$$

Using these series expansions, the expressions for $x_{h}$ and $y_{h}$ can be further rewritten as follows:

(13) $$x_{h}=x_{0}+\cos\gamma \cdot \sum_{n=0}^{\infty }\int_{0}^{L}\frac{{\left(-1\right)}^{n}\beta^{2n}}{(2n)!}\;dl-\sin\gamma \cdot \sum_{n=0}^{\infty }\int_{0}^{L}\frac{{\left(-1\right)}^{n}\beta^{2n+1}}{(2n+1)!}\;dl$$

(14) $$y_{h}=y_{0}+\sin\gamma \cdot \sum_{n=0}^{\infty }\int_{0}^{L}\frac{{\left(-1\right)}^{n}\beta^{2n}}{(2n)!}\;dl+\cos\gamma \cdot \sum_{n=0}^{\infty }\int_{0}^{L}\frac{{\left(-1\right)}^{n}\beta^{2n+1}}{(2n+1)!}\;dl$$

To simplify these expressions, we introduce the following notation:

(15) $$C_{n}=\int_{0}^{L}\frac{{\left(-1\right)}^{n}\beta^{2n}}{(2n)!}\;dl$$

(16) $$S_{n}=\int_{0}^{L}\frac{{\left(-1\right)}^{n}\beta^{2n+1}}{(2n+1)!}\;dl$$

Therefore, the simplified forms of the expressions are

(17) $$x_{h}=x_{0}+\cos\gamma \cdot \sum_{n=0}^{\infty }C_{n}-\sin\gamma \cdot \sum_{n=0}^{\infty }S_{n}$$

(18) $$y_{h}=y_{0}+\sin\gamma \cdot \sum_{n=0}^{\infty }C_{n}+\cos\gamma \cdot \sum_{n=0}^{\infty }S_{n}$$

For the clothoid curve, it is observed that the curvature *k* is a primary function of the arc length *l*, and the tangent angle θ is a quadratic function of the arc length *l*, as shown in Equation (2). Therefore, it is assumed that k(l) and θ(l) can be expressed in the following forms:

(19) k(l)=al+b

(20) $$\theta \left(l\right)=\frac{1}{2}al^{2}+bl+c$$

where *a* is the fixed parameter of the clothoid curve. By substituting l=0 into the equations, we obtain

(21) $$k\left(0\right)=k_{0}=b$$

(22) $$\theta \left(0\right)=\theta_{0}=c$$

That is, *b* is equal to the initial curvature $k_{0}$ of the curve, and *c* is euqal to the initial tangential angle $\theta_{0}$ of the curve. From this, the equation for the tangential angle θ(l) of the clothoid curve can be derived as follows:

(23) $$\begin{array}{cc} \theta (l) & =\frac{1}{2}al^{2}+k_{0}l+\theta_{0}\\ \; & ={\left(\sqrt{\frac{a}{2}}\cdot l+\frac{k_{0}}{\sqrt{2a}}\right)}^{2}+\left(\theta_{0}-\frac{k_{0}^{2}}{2a}\right) \end{array}$$

Furthermore, we can derive the following expressions for β(l) and γ:

(24) $$\beta \left(l\right)={\left(\sqrt{\frac{a}{2}}\cdot l+\frac{k_{0}}{\sqrt{2a}}\right)}^{2}$$

(25) $$\gamma =\left(\theta_{0}-\frac{k_{0}^{2}}{2a}\right)$$

By substituting the above formulas into Equations (15) and (16), we obtain the following expressions for $C_{n}$ and $S_{n}$:

(26) $$\begin{array}{cc} \; & C_{n}=\int_{0}^{L}\frac{{\left(-1\right)}^{n}{\left(\sqrt{\frac{a}{2}}\cdot l+\frac{k_{0}}{\sqrt{2a}}\right)}^{4n}}{(2n)!}\;dl\\ \; & \;\;\;=\frac{{\left(-1\right)}^{n}}{(2n)!}\int_{0}^{L}{\left(\sqrt{\frac{a}{2}}\cdot l+\frac{k_{0}}{\sqrt{2a}}\right)}^{4n}\;dl\\ \; & \;\;\;=\frac{{\left(-1\right)}^{n}}{(2n)!}\cdot \sqrt{\frac{2}{a}}\cdot \frac{1}{4n+1}\cdot {\left(\sqrt{\frac{a}{2}}\cdot l+\frac{k_{0}}{\sqrt{2a}}\right)}^{4n+1}{|}_{0}^{L}\\ \; & \;\;\;=\frac{{\left(-1\right)}^{n}\cdot \sqrt{\frac{2}{a}}}{(4n+1)(2n)!}\left[{\left(\sqrt{\frac{a}{2}}\cdot L+\frac{k_{0}}{\sqrt{2a}}\right)}^{4n+1}-{\left(\frac{k_{0}}{\sqrt{2a}}\right)}^{4n+1}\right] \end{array}$$

(27) $$\begin{array}{cc} \; & S_{n}=\int_{0}^{L}\frac{{\left(-1\right)}^{n}{\left(\sqrt{\frac{a}{2}}\cdot l+\frac{k_{0}}{\sqrt{2a}}\right)}^{4n+2}}{(2n+1)!}\cdot dl\\ \; & \;\;\;\;\;=\frac{{\left(-1\right)}^{n}}{(2n+1)!}\cdot \int_{0}^{L}{\left(\sqrt{\frac{a}{2}}\cdot l+\frac{k_{0}}{\sqrt{2a}}\right)}^{4n+2}\cdot dl\\ \; & \;\;\;\;\;=\frac{{\left(-1\right)}^{n}}{(2n+1)!}\cdot \sqrt{\frac{2}{a}}\cdot \frac{1}{4n+3}\cdot {\left(\sqrt{\frac{a}{2}}\cdot l+\frac{k_{0}}{\sqrt{2a}}\right)}^{4n+3}{|}_{0}^{L}\\ \; & \;\;\;\;\;=\frac{{\left(-1\right)}^{n}\cdot \sqrt{\frac{2}{a}}}{(4n+3)(2n+1)!}\left({\left(\sqrt{\frac{a}{2}}\cdot L+\frac{k_{0}}{\sqrt{2a}}\right)}^{4n+3}-{\left(\frac{k_{0}}{\sqrt{2a}}\right)}^{4n+3}\right) \end{array}$$

Thus, the coordinates $\left(x_{h},y_{h}\right)$ of any point on the clothoid curve can be expressed as a function of the independent variables *a*, $\theta_{0}$, $k_{0}$, and *L*. For a two-wheel differential robot, when docked at the initial station, the curvature is 0, and the azimuth of the starting point after preprocessing is also 0. By substituting γ=0 into Equation (25), the coordinates $\left(x_{h},y_{h}\right)$ of any point on the clothoid curve can be expressed as

(28) $$x_{h}=\sum_{n=0}^{N}\frac{{\left(-1\right)}^{n}a^{2n}L^{4n+1}}{\left(2n\right)!\left(4n+1\right)2^{2n}}$$

(29) $$y_{h}=\sum_{n=0}^{N}\frac{{\left(-1\right)}^{n}a^{2n+1}L^{4n+3}}{\left(2n+1\right)!\left(4n+3\right)2^{2n+1}}$$

## 3. Proposed Algorithm

### 3.1. Trajectory Generation Module

In this section, we focus on modeling the trajectory planning scheme based on clothoid curves. The proposed approach constructs a trajectory using a straight-line segment + composite curve structure, where the composite curve consists of clothoid segments and a circular arc. Due to the relative orientation difference between the initial and target stations, the trajectory must satisfy the heading angle constraints at the connection points. Therefore, we first design the composite curve to meet these angular requirements. The length of the straight segment can be easily computed by subtracting the coordinates once the composite curve is determined. Hence, we limit our discussion here to the modeling of the composite curve.

**Figure 3:**
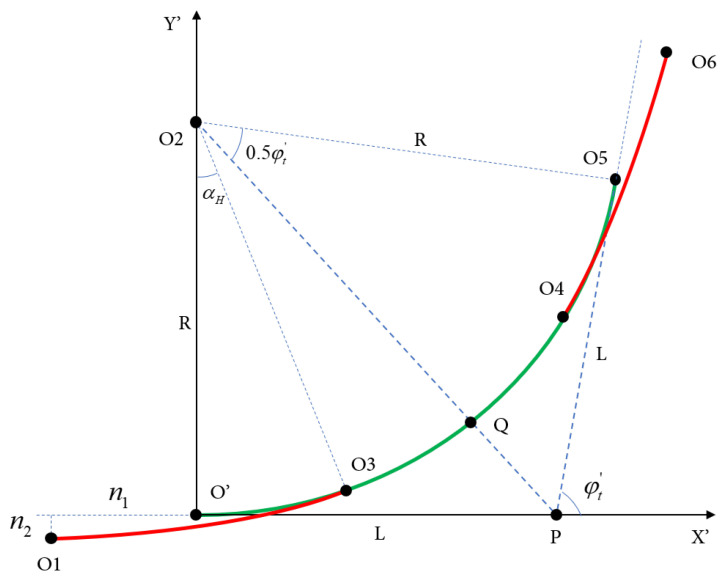
Theoretical model of the composite curve.

As shown in Figure 3, the green solid line represents the circular arc, while the red solid line denotes the clothoid segment. The composite curve starts at point $O_{1}$, where the tangent angle is zero, and it ends at point $O_{6}$, where the tangent angle is $\varphi_{t}^{\prime }$, representing the orientation difference between the start and target stations. The center of the circular arc is denoted by point $O_{2}$, and its radius is *R*. The clothoid is tangentially connected to the arc at point $O_{3}$.

The clothoid segment from $O_{1}$ to $O_{3}$ is symmetrically mirrored about the line segment $PO_{2}$, resulting in a mirrored clothoid curve from point *Q* to point $O_{6}$. The complete composite curve, composed of a clothoid–arc–clothoid structure, thus satisfies both the orientation constraints at the endpoints and the curvature continuity.

By adjusting the *a* parameter of the clothoid and the radius *R* of the arc, the horizontal and vertical coordinate differences between $O_{1}$ and $O_{6}$ can be modified, enabling the precise alignment of the curve with the desired initial and terminal positions.

Next, the clothoid curve segment from point $O_{1}$ to point $O_{3}$ is discussed. According to Formula (1), the parameter of the clothoid curve is denoted as *a*, the total length of the curve is $L_{s}$, and the azimuth angle of the mobile robot at point $O_{3}$ is $\alpha_{H}$. The variable $n_{1}$ represents the difference in the abscissa between point $O^{\prime }$ and point $O_{1}$, while $n_{2}$ is the difference in the ordinate between point $O^{\prime }$ and point $O_{1}$. The variable $X_{h}$ denotes the difference in the abscissa between point $O_{3}$ and point $O_{1}$, and $Y_{h}$ represents the difference in the ordinate between point $O_{3}$ and point $O_{1}$.

By introducing the above formula for the clothoid curve, we can obtain the following equations:

(30) $$L_{s}=\frac{1}{R\cdot a}$$

(31) $$X_{h}=x_{h}=\sum_{n=0}^{N}\frac{{\left(-1\right)}^{n}a^{2n}L_{s}^{4n+1}}{\left(2n\right)!\left(4n+1\right)2^{2n}}$$

(32) $$Y_{h}=y_{h}=\sum_{n=0}^{N}\frac{{\left(-1\right)}^{n}a^{2n+1}L_{s}^{4n+3}}{\left(2n+1\right)!\left(4n+3\right)2^{2n+1}}$$

(33) $$\alpha_{H}=\frac{1}{2R^{2}a}$$

(34) $$n_{1}=X_{h}-R\cdot \sin\left(\alpha_{H}\right)$$

(35) $$n_{2}=Y_{h}-R\left(1-\cos\left(\alpha_{H}\right)\right)$$

where *N* is the order of the approximation for the clothoid curve. For simplicity in the calculations, we adopt N=5 uniformly in this experiment. The radius *R* can be obtained by solving the following nonlinear equation:

(36) $$\left(n_{1}+R\cdot \tan\left(\frac{\varphi_{t}^{\prime }}{2}\right)\right)-L=0$$

Therefore, once the *a* parameter of the clothoid curve is specified, the radius *R* of the arc segment can be computed using the method described above. At this stage, all necessary parameters for constructing the composite *clothoid–arc–clothoid* curve are determined. The next step is to consider how to represent this combined curve using control points and the associated parameters.

As previously described, the clothoid segment begins at point $O_{1}$ and is tangent to the arc segment at point $O_{3}$. The curve then transitions into the arc segment from $O_{3}$ to $O_{4}$, followed by a second clothoid segment with identical parameters extending from $O_{4}$ to $O_{6}$. The full composite curve consists of a clothoid from $O_{1}$ to $O_{3}$, followed by an arc from $O_{3}$ to point *Q*, and it is then symmetrically reflected about the line segment $O_{2}P$. This reflection can be achieved using an affine transformation, with the transformation matrix denoted as T·M·F. Consequently, the coordinates of each control point along the trajectory, as well as the parameters of the respective curve segments, can be expressed through this affine mapping.

(37) $$F=\left[\begin{array}{ccc} -1 & 0 & 0\\ 0 & 1 & 0\\ 0 & 0 & 1 \end{array}\right],\;T=\left[\begin{array}{ccc} 1 & 0 & X_{t}\\ 0 & 1 & Y_{t}\\ 0 & 0 & 1 \end{array}\right]$$

(38) $$M=\left[\begin{array}{ccc} \cos\varphi_{t}^{\prime } & -\sin\varphi_{t}^{\prime } & 0\\ \sin\varphi_{t}^{\prime } & \cos\varphi_{t}^{\prime } & 0\\ 0 & 0 & 1 \end{array}\right]$$

(39) $$\left\{\begin{array}{c} O_{1}:{\left(\begin{array}{ccc} 0 & 0 & 1 \end{array}\right)}^{T}\\ O_{3}:{\left(\begin{array}{ccc} X_{h} & Y_{h} & 1 \end{array}\right)}^{T}\\ Q:{\left(\begin{array}{ccc} X_{h}+R\cdot \sin\frac{\varphi_{t}^{\prime }}{2}-R\cdot \sin\alpha_{H} & Y_{h}+R\cdot \cos\alpha_{H}-R\cdot \cos\frac{\varphi_{t}^{\prime }}{2} & 1 \end{array}\right)}^{T}\\ O_{4}:T\cdot M\cdot F\cdot \;Q\\ O_{6}:{\left(\begin{array}{ccc} X_{t}^{\prime } & Y_{t}^{\prime } & 1 \end{array}\right)}^{T} \end{array}\right.$$

(40) $$\left\{\begin{array}{c} C:\left(\begin{array}{cc} 0 & 0\\ k_{\max} & \alpha_{H} \end{array}\right|a_{\max}),\;O_{1}\to \;O_{3}\\ C:\left(\begin{array}{cc} k_{\max} & \alpha_{H}\\ k_{\max} & \varphi_{t}^{\prime }-\alpha_{H} \end{array}\right|0),\;O_{3}\to \;O_{4}\\ C:\left(\begin{array}{cc} k_{\max} & \varphi_{t}^{\prime }-\alpha_{H}\\ 0 & \varphi_{t}^{\prime } \end{array}\right|-a_{\max}),\;O_{4}\to \;O_{6} \end{array}\right.$$

The meanings of the parameters in $C:\left(\begin{array}{cc} k_{0} & \varphi_{0}\\ k_{t} & \varphi_{t} \end{array}\right|a)$ are as follows: $k_{0}$ is the curvature at the starting position, $\varphi_{0}$ is the azimuth at the starting position, $k_{t}$ is the curvature at the target position, $\varphi_{t}$ is the azimuth at the target position, and *a* is the curvature change rate of this curve. Additionally, *a* is the parameter of the clothoid curve, and for the arc curve, a=0.

At this stage, the composite clothoid–arc–clothoid curve has been fully constructed, and the coordinates of the control points, along with the parameters for each corresponding segment, can be stored. With these parameters, the planned trajectory can be readily reconstructed using the derived clothoid curve equations. Within this composite curve, the curvature of the arc segment is given by $k=\frac{1}{R}$. Since the curvature of a clothoid increases linearly with the arc length, $\frac{1}{R}$ also represents the maximum curvature $k_{\max}$ of the entire trajectory.

Assuming that the two-wheeled differential mobile robot moves with a uniform speed during the tracking of the entire combined curve, its maximum speed $v_{\max}$ must satisfy the following relation:

(41) $$v_{\max}^{2}\cdot k_{\max}=fg$$

where fg is the safety factor set during the operation of the two-wheeled differential mobile robot. After the *a* parameter is given and the maximum curvature $k_{\max}$ is calculated, the total running time of the two-wheeled differential mobile robot is also determined by the following equation:

(42) $$T_{\mathrm{total}}=\frac{2L_{s}+R\left(\varphi_{t}^{\prime }-2\alpha_{H}\right)+S_{1}+S_{2}-2L}{v_{\max}}$$

where $2L_{s}+R\left(\varphi_{t}^{\prime }-2\alpha_{H}\right)$ represents the total length of the combined curve, and $S_{1}+S_{2}-2L$ is the translation length of the starting point or the target point, which is considered the linear motion of the mobile robot.

**Figure 4:**
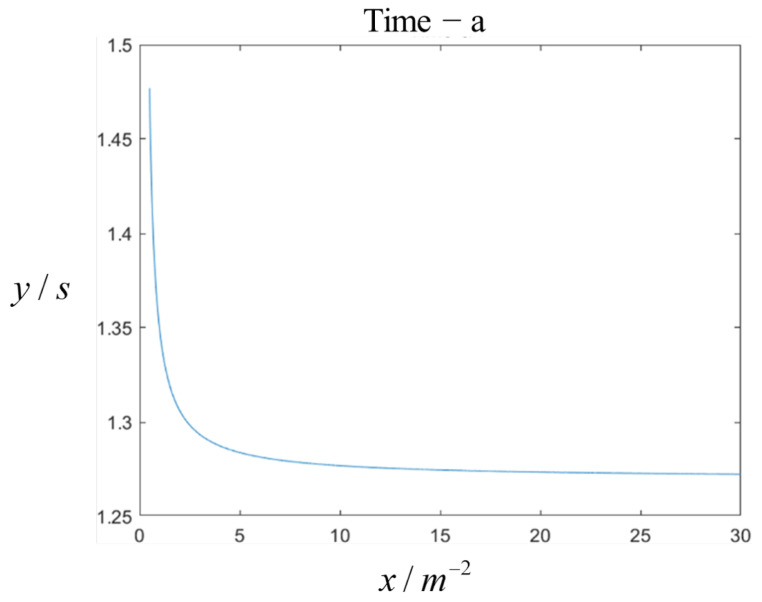
Theoretical model of the clothoid curve.

So far, a transition curve consisting of a clothoid curve–arc curve–clothoid curve has been constructed between the starting point and the target point after the coordinate transformation preprocessing. After the complete planning of the entire path, it is necessary to further discuss the influence of the clothoid curve’s parameter setting on the running time and trajectory. By solving Equation (36), the correspondence between the parameters can be obtained. Based on this, the running time becomes a function related only to the variable, allowing for a theoretical analysis of the monotonicity of the running time with respect to this variable. Experimental verification demonstrates that there is a monotonically decreasing relationship between the parameter and the running time: as the given parameter of the clothoid curve increases, the total running time of the entire path decreases. The results of a Matlab simulation and verification experiment are shown in Figure 4, with the parameters in the figure ranging from 0.5 m to 30 m. This relationship is based on the initial point, azimuth angle, target point, safety factor, and target azimuth angle. Since the physical conditions are not strictly limited and the actual two-wheeled differential mobile robot needs to undergo acceleration and deceleration phases, this conclusion can only serve as a reference for selecting the curvature change rate parameters.

### 3.2. Kinematic Modeling of Two-Wheel Differential Robot

Given that the curvature of a clothoid curve changes linearly with the path length, we consider a four-wheeled vehicle as an example. In this case, if the vehicle maintains a constant linear speed while turning and its steering wheel rotates at a uniform rate, the resulting trajectory follows a clothoid curve. This practical analogy highlights a significant limitation of the clothoid curve when applied to a two-wheeled differential-drive robot.

Unlike four-wheeled vehicles, a two-wheeled differential-drive robot relies on the independent speed control of its two wheels for both steering and forward motion. Since turning is achieved through the speed ratio between the two wheels and the overall velocity is also jointly determined by them, steering and forward movement are inherently coupled. Consequently, changes in direction necessitate variations in wheel speed, which are constrained by physical limits on velocity and acceleration.

If the curvature change rate of the clothoid curve is too large, the planned trajectory may violate the kinematic constraints of the robot, making it infeasible to execute. Therefore, it is essential to impose restrictions and optimize the parameter settings to ensure that the planned trajectory remains within the robot’s operational limits.

**Figure 5:**
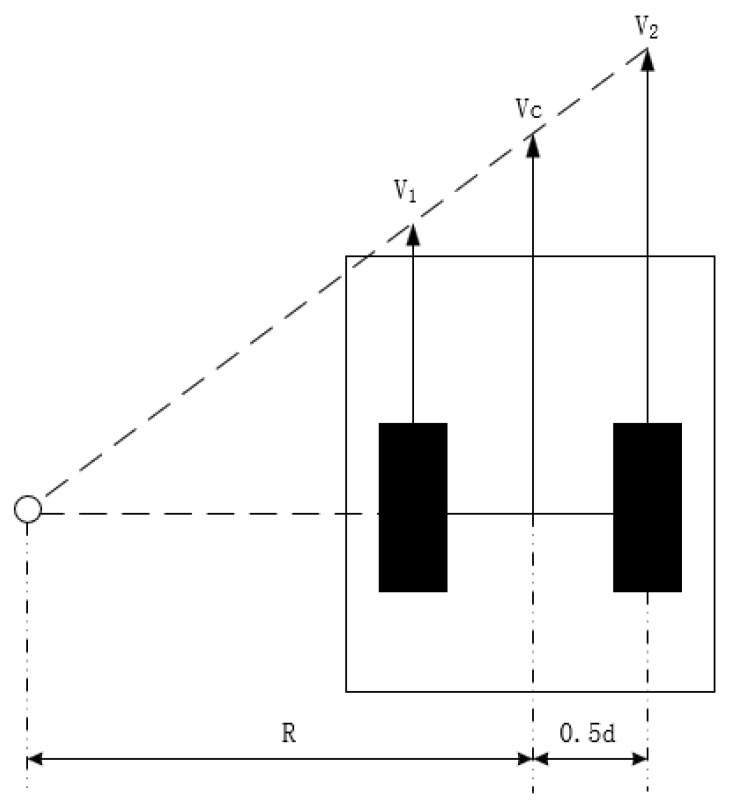
Kinematics model of two-wheel differential robot.

Assume that a two-wheeled differential-drive robot executes a left-turn maneuver. Let the speed of the left wheel be $v_{1}$, the speed of the right wheel be $v_{2}$, the wheelbase (the distance between the two wheels) be *d*, the linear velocity of the robot be $v_{c}$, and the turning radius be *R*, as shown in Figure 5. The turning radius and linear velocity can be expressed in terms of $v_{1}$, $v_{2}$, and *d* as follows:

(43) $$R=\frac{(v_{1}+v_{2})d}{2(v_{2}-v_{1})}$$

(44) $$v_{c}=\frac{1}{2}\left(v_{1}+v_{2}\right)$$

The curvature *k* of the trajectory is then given by

(45) $$k=\frac{1}{R}=\frac{2}{d}\cdot \frac{v_{2}-v_{1}}{v_{2}+v_{1}}$$

Since parameter *a* of the clothoid curve characterizes the rate of curvature change with respect to the arc length, it is necessary to compute the derivative of the curvature $\frac{dk}{dx}$. For convenience, we denote this derivative as $k^{\prime }$.

(46) $$k^{\prime }=\frac{dk}{dx}=-\frac{4}{d}\cdot \frac{\frac{dv_{1}}{dx}\cdot v_{2}-v_{1}\cdot \frac{dv_{2}}{dx}}{{\left(v_{1}+v_{2}\right)}^{2}}=-\frac{4}{d}\cdot \frac{v_{1}^{\prime }v_{2}-v_{1}v_{2}^{\prime }}{{\left(v_{1}+v_{2}\right)}^{2}}$$

where $v_{1}^{\prime }$ and $v_{2}^{\prime }$ denote the derivatives of $v_{1}$ and $v_{2}$ with respect to the path length. If the accelerations of the left and right wheels are denoted as $m_{1}$ and $m_{2}$, respectively, then $v_{1}^{\prime }$ and $v_{2}^{\prime }$ are given by

(47) $$v_{1}=\sqrt{2m_{1}x+v_{10}^{2}}$$

(48) $$\frac{dv_{1}}{dx}=\frac{m_{1}}{\sqrt{2m_{1}x+v_{10}^{2}}}=\frac{m_{1}}{v_{1}}$$

Using these expressions, $k^{\prime }$ can be rewritten in terms of $v_{1}$, $v_{2}$, $m_{1}$, and $m_{2}$. By setting $k^{\prime }=a$, we obtain the relationship between the accelerations of the left and right wheels:

(49) $$m_{1}=\frac{{v_{1}}^{2}}{{v_{2}}^{2}}m_{2}-\frac{ad}{4}\cdot \frac{v_{1}}{v_{2}}\cdot {\left(v_{1}+v_{2}\right)}^{2}$$

This equation implies that, when the robot follows a clothoid curve during turning, the accelerations of the left and right wheels must satisfy a specific relationship. Moreover, this relationship depends not only on the accelerations but also on the current speeds of the wheels.

### 3.3. Velocity Constraints in the Critical State

**Figure 6:**
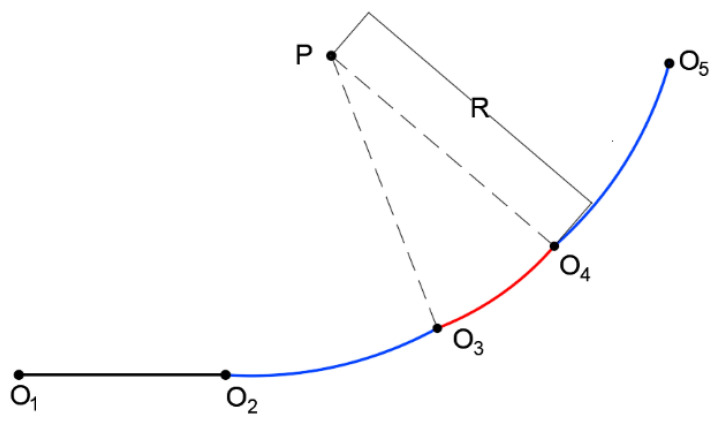
Velocity planning node.

To further analyze a complete trajectory, we consider a scenario in which the two-wheeled differential robot initially moves in a straight line before executing a turn, with its velocity at points $O_{1}$ and $O_{5}$ being zero. The planned trajectory for this case is illustrated in Figure 6.

Point $O_{1}$ is the starting point, and point $O_{5}$ is the endpoint. The trajectory consists of four segments: the $O_{1}$-$O_{2}$ segment is a straight line, the $O_{2}$-$O_{3}$ segment is a clothoid transition curve, the $O_{3}$-$O_{4}$ segment is an arc, and the $O_{4}$-$O_{5}$ segment is a symmetrical clothoid transition curve.

When the two-wheeled differential-drive robot reaches point $O_{2}$ through linear motion, its wheel velocities satisfy $v_{1}=v_{2}$. At this point, the robot enters the clothoid segment, where the accelerations of the left and right wheels must satisfy Equation (49). Substituting $v_{1}=v_{2}=v_{O2}$ into this equation yields the following:

(50) $$m_{1}=m_{2}-adv_{O2}^{2}$$

Since *a*, *d*, and $v_{O2}$ are all non-negative, it follows that $m_{1}\leq m_{2}$. When $adv_{O2}^{2}$ is large, $m_{1}$ decreases, and, given that the wheel acceleration of the two-wheeled differential-drive robot cannot be infinite, the constraint

(51) $$-m_{\max}\leq m_{1},m_{2}\leq m_{\max}$$

must be satisfied. To ensure that the robot adheres to this acceleration constraint at point $O_{2}$, we obtain the following:

(52) $$-m_{\max}\leq m_{2}-adv_{O2}^{2}$$

(53) $$v_{O2}\leq \sqrt{\frac{2m_{1}}{ad}}\leq \sqrt{\frac{2m_{\max}}{ad}}$$

During the execution of the clothoid curve trajectory, if there exists a velocity pair $v_{1},v_{2}$ and a corresponding acceleration $m_{2}$ such that $m_{1}=-m_{\max}$, the system is said to be in a critical state. If $m_{2}<m_{\max}$, increasing $m_{2}$ allows $m_{1}$ to exceed $-m_{\max}$, thereby escaping the critical state. However, if $m_{2}$ gradually increases until it reaches $m_{\max}$, no further adjustments can be made, and the system remains in the critical state. At this point, with $m_{2}=m_{\max}$ and $m_{1}=-m_{\max}$, after an infinitesimal time interval dt, the system satisfies the following:

(54) $$m_{1}=\frac{v_{1}^{2}}{v_{2}^{2}}m_{\max}-\frac{ad}{4}\cdot \frac{v_{1}}{v_{2}}\cdot {\left(v_{1}+v_{2}\right)}^{2}=-m_{\max}$$

(55) $$\begin{array}{cc} & m_{1}=\frac{{\left(v_{1}-m_{\max}dt\right)}^{2}}{{\left(v_{2}+m_{\max}dt\right)}^{2}}m_{\max}-\frac{ad}{4}\cdot \frac{v_{1}-m_{\max}dt}{v_{2}+m_{\max}dt}\cdot {\left(v_{1}+v_{2}\right)}^{2}\\ & =\frac{{\left(v_{1}-m_{\max}dt\right)}^{2}m_{\max}-\frac{ad}{4}\left(v_{1}-m_{\max}dt\right)\left(v_{2}+m_{\max}dt\right){\left(v_{1}+v_{2}\right)}^{2}}{{\left(v_{2}+m_{\max}dt\right)}^{2}}\\ & =\frac{\left(\left(v_{1}^{2}+m_{\max}^{2}dt^{2}-2v_{1}m_{\max}dt\right)m_{\max}-\frac{ad}{4}\cdot {\left(v_{1}+v_{2}\right)}^{2}\left(v_{1}v_{2}+\left(v_{1}-v_{2}\right)m_{\max}dt-m_{\max}^{2}dt^{2}\right)\right)}{{\left(v_{2}+m_{\max}dt\right)}^{2}}\\ & =\frac{\left(v_{1}^{2}m_{\max}-\frac{ad}{4}{\left(v_{1}+v_{2}\right)}^{2}v_{1}v_{2}\right)+\left(m_{\max}dt^{2}-2v_{1}dt\right)m_{\max}^{2}}{{\left(v_{2}+m_{\max}dt\right)}^{2}}\\ & \;-\frac{\frac{ad}{4}{\left(v_{1}+v_{2}\right)}^{2}\left(\left(v_{1}-v_{2}\right)m_{\max}dt-m_{\max}^{2}dt^{2}\right)}{{\left(v_{2}+m_{\max}dt\right)}^{2}}\\ & =\frac{v_{2}^{2}}{{\left(v_{2}+m_{\max}dt\right)}^{2}}\left(-m_{\max}\right)\\ & \;+\frac{m_{\max}^{2}}{{\left(v_{2}+m_{\max}dt\right)}^{2}}\left(\left(m_{\max}dt^{2}-2v_{1}dt\right)-\frac{v_{2}}{v_{1}}\left(\frac{v_{1}^{2}}{v_{2}^{2}}+1\right)\left(\left(v_{1}-v_{2}\right)dt-m_{\max}dt^{2}\right)\right)\\ & =\frac{-m_{\max}}{{\left(v_{2}+m_{\max}dt\right)}^{2}}\left(v_{2}^{2}-\left(m_{\max}^{2}dt^{2}-2v_{1}m_{\max}dt\right)+\frac{v_{2}}{v_{1}}\left(\frac{v_{1}^{2}}{v_{2}^{2}}+1\right)\left(\left(v_{1}-v_{2}\right)m_{\max}dt-m_{\max}^{2}dt^{2}\right)\right)\\ & =\frac{-m_{\max}}{{\left(v_{2}+m_{\max}dt\right)}^{2}}\left(v_{2}^{2}-m_{\max}^{2}dt^{2}+2v_{1}m_{\max}dt+\left(\frac{v_{2}}{v_{1}}+\frac{v_{1}}{v_{2}}\right)\left(\left(v_{1}-v_{2}\right)m_{\max}dt-m_{\max}^{2}dt^{2}\right)\right)\\ & =-m_{\max}\left(1+\frac{\left(2m_{\max}+\frac{v_{1}^{2}+v_{2}^{2}}{v_{1}v_{2}}\right)\left(-m_{\max}dt-v_{2}+v_{1}\right)m_{\max}dt}{{\left(v_{2}+m_{\max}dt\right)}^{2}}\right) \end{array}$$

Since $v_{1}<v_{2}$, the above equation holds only at dt=0. For any dt>0, the right-hand side is greater than $-m_{\max}$, meaning that the system naturally escapes the critical state. Therefore, concerns about acceleration constraints along the clothoid curve trajectory are unnecessary, and only the acceleration at the starting point (where the curvature is zero) needs to be considered.

Once the constraint on the curvature change rate parameter of the clothoid curve is determined, the velocity profile of the two-wheeled differential-drive robot along the entire trajectory can be planned efficiently, allowing for the rapid generation of feasible motion plans.

### 3.4. Velocity Planning of Two-Wheel Differential Robot

In the clothoid curve model, the linear velocities along the trajectory from $O_{1}$ to $O_{5}$ are denoted as $v_{c1}$ to $v_{c5}$, while the corresponding velocities of the left and right wheels are represented by $v_{11}$ to $v_{15}$ and $v_{21}$ to $v_{25}$, respectively. The complete trajectory consists of consecutive segments: a straight line, a clothoid curve, an arc segment, and another clothoid curve, starting from $O_{1}$. In some cases, the trajectory may begin directly with a clothoid curve instead of a straight segment. When this occurs, the planning sequence can be adjusted accordingly, allowing trajectory generation to start from the target point. This adjustment facilitates the construction of a more unified mathematical model.

The velocity of the arc segment is primarily determined by the velocities at points $O_{3}$ and $O_{4}$, making it essential to consider these speeds first in the velocity planning process. Once these velocities are determined, the feasibility and optimality of the velocities at other points should be evaluated. Typically, a mobile robot travels faster along straight-line segments and must decelerate when approaching curves to avoid instability or the risk of rollover caused by excessive speed. Therefore, to minimize the total traversal time, the robot should maintain the maximum allowable speed at each point while ensuring safety.

At point $O_{3}$, the velocities of the left and right wheels of a two-wheeled differential robot are computed based on safety constraints. The maximum allowable linear velocity at $O_{3}$ is determined by

(56) $$v_{c3\max}=\sqrt{R\cdot f\cdot g}$$

From Equation (43), the expressions for $v_{13}$ and $v_{23}$ can be derived accordingly.

(57) $$\begin{array}{c} \left\{\begin{array}{c} v_{23\max}=v_{c3\max}\left(\frac{d}{2R}+1\right)\\ v_{13\max}=\frac{4v_{23\max}}{\frac{d}{R}+2}-v_{23\max}\\ v_{23}=\min\left(v_{\max},v_{23\max}\right)\\ v_{13}=\frac{4v_{23}}{\frac{d}{R}+2}-v_{23} \end{array}\right. \end{array}$$

The above derivation indicates that the two-wheeled differential robot can achieve the maximum possible linear velocity at point $O_{3}$. Once the velocities of the two wheels at $O_{3}$ are determined, it is further necessary to consider how to set the acceleration of the wheels along the clothoid curve trajectory. Given the objective of minimizing the travel time from $O_{2}$ to $O_{3}$, the fundamental kinematic equation

(58) $$x=vt+\frac{1}{2}at^{2}$$

suggests that, for a given distance and velocity, increasing the acceleration reduces the travel time. Since the velocity at $O_{3}$ is predetermined, the linear velocity should be maximized throughout the transition from $O_{2}$ to $O_{3}$. This implies that the left wheel of the robot remains in a deceleration state throughout this process.

Denoting the average acceleration of the left wheel during the movement from $O_{2}$ to $O_{3}$ as $m_{e}$, the following relationship between distance and time can be established:

(59) $$x=v_{13}t-\frac{1}{2}m_{e}t^{2}$$

From this, it follows that the travel time *t* is inversely related to the average acceleration $m_{e}$. Therefore, to minimize the travel time, $m_{e}$ should be as small as possible, leading to the choice $m_{e}=-m_{\max}$. Given that $m_{1}\geq -m_{\max}$, the above equation holds only if $m_{1}\equiv -m_{\max}$.

Thus, the velocities of the left and right wheels at $O_{2}$ can be computed as follows:

(60) $$v_{12}=v_{22}=\sqrt{v_{13}^{2}+2m_{\max}L_{\mathrm{left}}}$$

where $L_{\mathrm{left}}$ represents the path length of the left wheel in the clothoid curve segment.

In order to obtain $L_{\mathrm{left}}$, it is necessary to analyze the relationship between the velocities of the left and right wheels and the linear velocity of the two-wheeled differential robot. The specific derivation process is as follows:

(61) $$\begin{array}{cc} \; & k=\frac{2}{d}\cdot \frac{v_{2}-v_{1}}{v_{2}+v_{1}},\\ \; & v_{2}=\frac{4v_{1}}{2-kd}-v_{1},\\ \; & v_{c}=\frac{1}{2}\left(v_{1}+v_{2}\right),\\ \; & dL_{1}=v_{1}\cdot dt,\\ \; & dL_{c}=v_{c}\cdot dt=\frac{1}{2}\left(v_{1}+v_{2}\right)dt,\\ \; & \Rightarrow \frac{dL_{c}}{dL_{1}}=\frac{\frac{1}{2}\left(v_{1}+v_{2}\right)}{v_{1}}=\frac{2}{2-kd}. \end{array}$$

The above equations establish the ratio between the micro-components of the left wheel displacement and the overall movement of the two-wheeled differential robot. Specifically, in the clothoid curve segment, given that

(62) $$k=a\cdot L_{c},$$

the expression for $L_{\mathrm{left}}$ can be derived by substituting the above equation and applying calculus principles:

(63) $$\begin{array}{cc} \; & \frac{dL_{c}}{dL_{1}}=\frac{2}{2-adL_{c}},\\ \; & \left(1-\frac{adL_{c}}{2}\right)dL_{c}=dL_{1},\\ \; & \Rightarrow L_{1}=L_{c}-\frac{1}{4}adL_{c}^{2}+L_{0}. \end{array}$$

When $L_{c}=0$, we have $L_{1}=0$, which leads to

(64) $$L_{1}=L_{c}-\frac{1}{4}adL_{c}^{2}.$$

Since the clothoid curve segment satisfies $L_{c}=L_{s}$, it follows that

(65) $$L_{\mathrm{left}}=L_{s}-\frac{1}{4}adL_{s}^{2}.$$

At this point, the velocities of the two wheels at point $O_{2}$ have also been determined.

Through the above derivation, it can be observed that the velocity of the two-wheeled differential robot at point $O_{2}$ is greater than zero, whereas the linear velocity at point $O_{5}$ is exactly zero. This results in an asymmetry between the speed planning of the clothoid curve segments $O_{2}$-$O_{3}$ and $O_{4}$-$O_{5}$.

Similar to the segment $O_{2}$-$O_{3}$, the segment $O_{4}$-$O_{5}$ should ensure that the left wheel undergoes maximum deceleration at point $O_{5}$. Consequently, the velocity at $O_{4}$ can be derived from the velocity at $O_{5}$:

(66) $$v_{14}=v_{24}=\sqrt{2m_{\max}L_{\mathrm{left}}}.$$

Since there is no explicit size relationship between $v_{13}$ and $v_{14}$, it is necessary to determine the required operations for the two-wheeled differential robot along the arc curve segment $O_{3}$-$O_{4}$ in order to meet the velocity constraints at $O_{4}$. Given that the initial velocity, target velocity, and path length along the arc curve are known, a physical analysis suggests that a higher acceleration results in a shorter traversal time.

Therefore, the shortest travel time can be achieved by setting the acceleration of the right wheel to $m_{2}\equiv m_{\max}$ in the variable-speed motion along the arc when $v_{13}<v_{14}$ and by setting the acceleration of the left wheel to $m_{1}\equiv -m_{\max}$ when $v_{13}>v_{14}$.

On the straight-line segment $O_{1}$-$O_{2}$, the left and right wheels maintain the same velocity and acceleration. A physical analysis shows that the acceleration along this segment should be

(67) $$m_{1}\equiv m_{2},$$

which corresponds to either the maximum or minimum acceleration required for the shortest travel time. At this point, the velocity at each key point and the acceleration profile for each segment of the two-wheeled differential robot’s trajectory have been determined, allowing for the next step: the construction of an optimization model.

### 3.5. Optimization Module


(68) $$\left\{\begin{array}{c} v_{11}=v_{21}=0\\ v_{12}=v_{22}=\sqrt{v_{13}^{2}+2m_{\max}L_{\mathrm{left}}}\\ \left\{\begin{array}{c} v_{13}=\frac{4v_{23}}{\frac{d}{R}+2}-v_{23}\\ v_{23}=\min\left(v_{\max},v_{23\max}\right) \end{array}\right.\\ \left\{\begin{array}{c} v_{14}=\sqrt{2m_{\max}L_{\mathrm{left}}}\\ v_{24}=\frac{4v_{14}}{2-\frac{d}{R}}-v_{14} \end{array}\right.\\ v_{15}=v_{25}=0 \end{array}\right.$$

In addition, the left wheel acceleration $m_{1}\equiv m_{\max}$ is maintained during the acceleration process along the straight line, and the left wheel acceleration $m_{1}\equiv -m_{\max}$ is maintained during the deceleration process, as well as in the clothoid curve segment. In the variable-speed process of the arc curve segment, different accelerations are selected based on the relationship between $v_{13}$ and $v_{14}$:

(69) $$\left\{\begin{array}{cc} m_{1}\equiv -m_{\max}, & \mathrm{if}\;v_{13}>v_{14}\\ m_{2}\equiv m_{\max}, & \mathrm{if}\;v_{13}<v_{14} \end{array}\right.$$

It is assumed that the shortest path for the two-wheeled differential robot to accelerate from 0 to $v_{\max}$ in a straight-line segment is $L_{\max}$, and the shortest path for the robot to accelerate from 0 to $v_{12}$ is $L_{jia}$. Based on the relationship between these two distances, different strategies should be chosen for the line segment’s motion process. The core strategy is to accelerate directly from point $O_{1}$ to the maximum speed $v_{\max}$ supported by the car and then to decelerate to point $O_{2}$ after traveling a distance at a constant speed. Since the length of the straight track $L_{0}$ may not allow the car to reach the maximum speed, the above core strategy can be expressed as follows:

(70) $$\begin{array}{cc} & \mathrm{if}\;L_{0}\geq 2L_{\max}-L_{jia}\\ & \;L_{1}=L_{\max}\\ & \;L_{2}=L_{0}-L_{1}-L_{2}\\ & \;L_{3}=L_{\max}-L_{jia}\\ & \mathrm{if}\;L_{0}<2L_{\max}-L_{jia}\;\mathrm{and}\;L_{0}\geq L_{jia}\\ & \;L_{1}=\frac{1}{2}\left(L_{0}+L_{jia}\right)\\ & \;L_{2}=0\\ & \;L_{3}=\frac{1}{2}\left(L_{0}-L_{jia}\right)\\ & \mathrm{if}\;L_{0}<L_{jia}\\ & \;\mathrm{error} \end{array}$$

According to the varying speeds of the two-wheel differential robot at points $O_{3}$ and $O_{4}$, distinct acceleration and deceleration strategies must be employed. The core objective is to maximize the portion of the trajectory traveled at the maximum allowable speed. The primary approach involves accelerating the robot as quickly as possible to reach this maximum speed.

Assuming that the left wheel follows a circular arc, where it accelerates over a distance $L_{4}$, maintains a constant speed over $L_{5}$, and decelerates over $L_{6}$, the process can be formulated as follows:

(71) $$\begin{array}{cc} & \mathrm{if}\;v_{13}<v_{14}\\ & \;L_{4}=\frac{v_{14}^{2}-v_{13}^{2}}{2m_{\max}}\\ & \;L_{5}=L_{r}-L_{4}\\ & \;L_{6}=0\\ & \mathrm{else}\\ & \;L_{4}=0\\ & \;L_{6}=\frac{v_{13}^{2}-v_{14}^{2}}{2m_{\max}}\\ & \;L_{5}=L_{r}-L_{6}\\ & \mathrm{end} \end{array}$$

where $L_{r}$ represents the length of the circular arc, given by

(72) $$L_{r}=R\varphi_{t}^{\prime }-\frac{1}{Ra}$$

where $\varphi_{t}^{\prime }$ is the target azimuth after preprocessing the initial conditions. At this point, the velocity at each point along the trajectory is explicitly defined, allowing for the computation of the total traversal time, $T_{\mathrm{total}}$, as follows:

(73) $$\begin{array}{cc} & T_{1}=\sqrt{\frac{2L_{1}}{m_{\max}}}\\ & T_{2}=\frac{L_{2}}{\sqrt{2m_{\max}L_{1}}}\\ & T_{3}=\frac{\sqrt{2m_{\max}L_{1}}-v_{12}}{m_{\max}}\\ & T_{4}=\frac{v_{12}-v_{13}}{m_{\max}}\\ & \mathrm{if}\;v_{13}<v_{14}\\ & \;T_{5}=\frac{v_{14}-v_{13}}{m_{\max}}\\ & \;T_{6}=\frac{L_{5}}{v_{14}}\\ & \;T_{7}=0\\ & \mathrm{else}\\ & \;T_{5}=0\\ & \;T_{6}=\frac{L_{5}}{v_{13}}\\ & \;T_{7}=\frac{v_{13}-v_{14}}{m_{\max}}\\ & \mathrm{end} \end{array}$$

The total traversal time is then obtained as

(74) $$T_{\mathrm{total}}=T_{1}+T_{2}+T_{3}+T_{4}+T_{5}+T_{6}+T_{7}$$

At this stage, the objective function for minimizing the total traversal time can be formulated. The next step involves optimizing the selection of the curvature rate parameter of the cyclotron curve using an optimization algorithm. The specific optimization model is formulated as

(75) $$\begin{array}{cc} & \min\;T_{\mathrm{total}}\\ & s.t.\;\left\{\begin{array}{c} -R\varphi_{t}^{\prime }+\frac{1}{Ra}<0,\\ L_{\mathrm{jia}}-L_{0}<0,\\ -2m_{\max}+ad\cdot v_{12}^{2}<0,\\ v_{12}-v_{\max}<0,\\ -L_{\mathrm{left}}<0. \end{array}\right. \end{array}$$

## 4. Experimentation

The experiment sets the simulation parameters according to the actual physical parameters of the two-wheeled differential-drive robot in the experimental field. Specifically, the parameters are

$$v_{\max}=1.2\;m/s,\;m_{\max}=1\;{m/s}^{2},\;d=0.6\;m,\;fg=6\;{m/s}^{2}.$$

**Figure 7:**
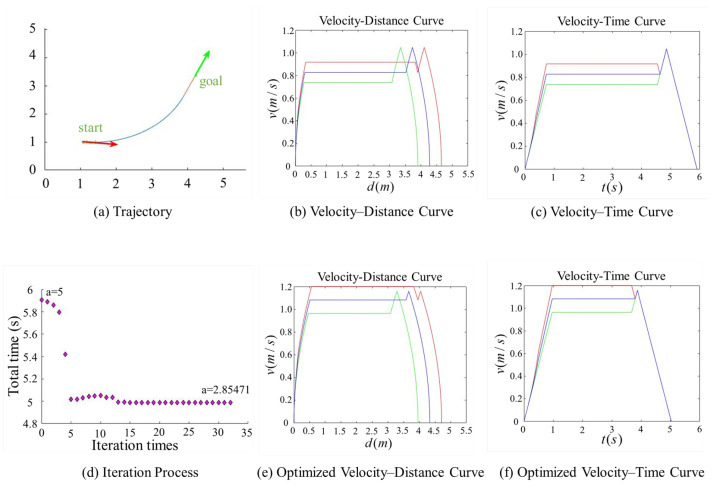
Comparison of trajectory performance for a path with an acute turning angle, where the robot fails to reach the maximum allowable velocity. In the plot, the red and green lines denote the velocities of the right and left wheels, respectively, and the blue line represents the robot’s linear velocity. The trajectory parameter *a* was initially set to 5 and was optimized to 2.85471.

**Figure 8:**
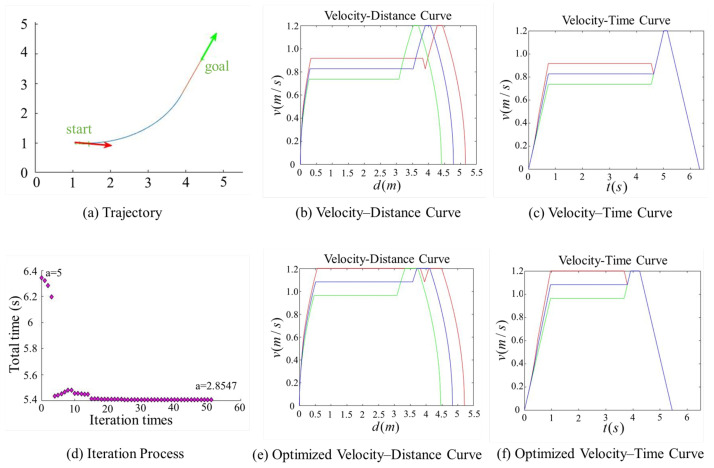
Comparison of trajectory performance for a path with an acute turning angle, where the robot successfully reaches the maximum allowable velocity. The goal point in this figure was generated by translating the goal point in Figure 7 by 0.5 m along the heading direction of its pose. The trajectory parameter *a* was initially set to 5 and was optimized to 2.8547.

**Figure 9:**
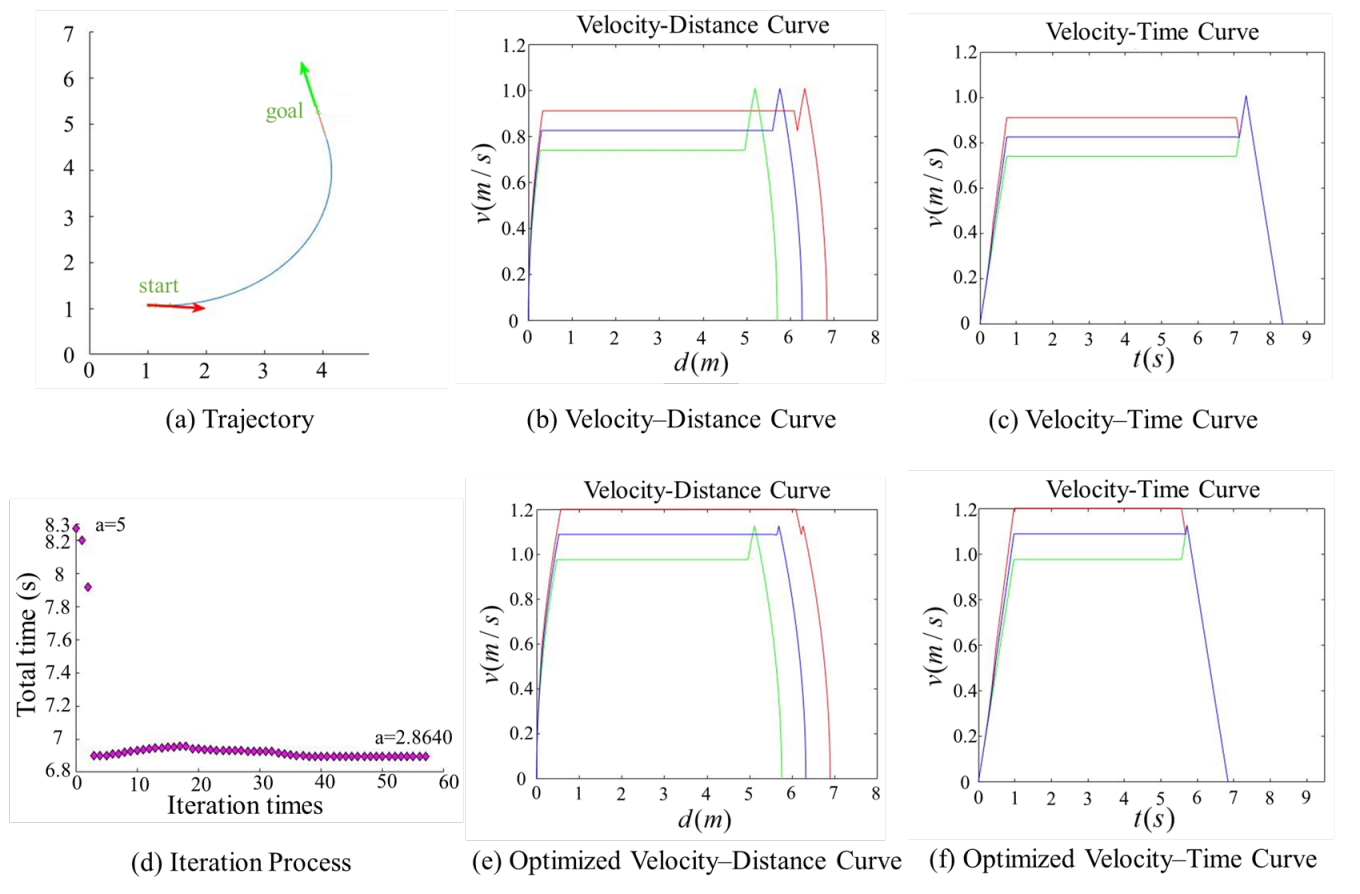
Comparison of trajectory performance for a path with an obtuse turning angle, where the robot fails to reach the maximum allowable velocity. The trajectory parameter *a* was initially set to 5 and was optimized to 2.8640.

**Figure 10:**
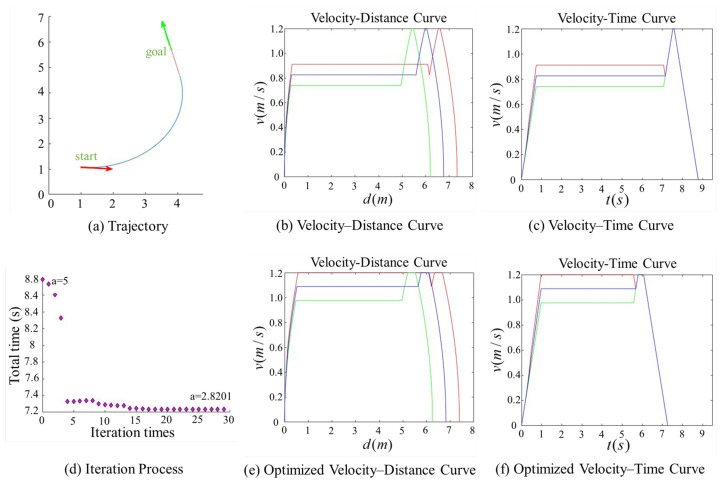
Comparison of trajectory performance for a path with an obtuse turning angle, where the robot successfully reaches the maximum allowable velocity. The goal point in this figure was generated by translating the goal point in Figure 9 by 0.5 m along the heading direction of its pose. The trajectory parameter *a* was initially set to 5 and was optimized to 2.8201.

Figure 7, Figure 8, Figure 9 and Figure 10 present comparison graphs of the optimization algorithms under different scenarios, where the distance is measured in meters, the time is measured in seconds, and the speed is measured in meters per second. Due to the strong correlation between the optimization effect and the actual path, the degree of optimization cannot be precisely quantified. Based on the experimental comparison diagram, it is evident that the two-wheel differential robot achieves a higher linear velocity on the optimized trajectory, particularly along the clothoid curve and arc segments, while consistently maintaining high acceleration. Specifically, the initial pose in Figure 8 is consistent with that in Figure 7, while the target position is obtained by shifting the target point in Figure 7 forward by 0.5 m along its orientation. A comparison between Figure 7 and Figure 8 reveals that, due to the shorter straight-line segment in Figure 7, the robot must begin decelerating before reaching the maximum velocity $v_{\max}$, thus limiting the potential for higher speeds. Although the maximum velocity is still not fully achieved after parameter optimization, both the peak velocity and the average velocity are improved.

In contrast, Figure 8 features an extended straight-line segment, which provides the robot with a sufficient acceleration distance to reach $v_{\max}$. Moreover, after parameter optimization, the robot is able to maintain this maximum speed for a longer period, thereby increasing the overall average velocity. Figure 9 and Figure 10 illustrate scenarios involving obtuse turning angles, under the same experimental setup as in Figure 7 and Figure 8. These comparative experiments demonstrate that, after trajectory parameter optimization, the robot is capable of sustaining high-speed motion for a longer duration, which significantly improves the average velocity along the trajectory and effectively reduces the task execution time. We conducted additional comparative experiments but only present the most representative cases here. The results show that, after parameter optimization, the overall execution time is reduced by approximately 15%.

## 5. Conclusions

In this study, an optimized steering trajectory planning algorithm based on clothoid curves is proposed for two-wheeled differential-drive robots. The method addresses common challenges such as prolonged execution times and difficulties in satisfying dynamic constraints. By constructing a nonlinear optimization model to regulate curvature variation, the proposed approach ensures feasible trajectories while improving overall execution efficiency. The main conclusions are as follows:

1. The mathematical formulation of the clothoid curve is simplified by leveraging the static characteristics of two-wheeled differential robots. This enables the generation of curvature-continuous trajectories, thereby enhancing motion smoothness and control stability.
2. A trajectory optimization algorithm is designed based on the kinematic characteristics of the robot, achieving a 15% reduction in the execution time and improving operational efficiency.

Experimental results demonstrate that the proposed algorithm not only generates feasible velocity commands that meet dynamic constraints but also significantly improves time efficiency compared to traditional methods. On the one hand, the algorithm enhances trajectory execution performance. On the other hand, its adaptability to dynamic environments remains limited. Future work will focus on integrating real-time perception and dynamic trajectory replanning mechanisms to improve responsiveness to moving or newly emerging obstacles. Moreover, further optimization is needed to enhance computational efficiency.

## Data Availability

The original contributions presented in the study are included in the article. Further inquiries can be directed to the corresponding author.

## References

[1] Correll N., Bekris K.E., Berenson D., Brock O., Causo A., Hauser K., Okada K., Rodriguez A., Romano J.M., Wurman P.R. (2018). Analysis and Observations From the First Amazon Picking Challenge. IEEE Trans. Autom. Sci. Eng..

[2] Marco A.C.-C., Victor A.-R., Uriel H.H.-B. (2015). Mobile robot path planning using artificial bee colony and evolutionary programming. Appl. Soft Comput..

[3] Zafar M.N., Mohanta J.C. (2018). Methodology for path planning and optimization of mobile robots: A review. Procedia Comput. Sci..

[4] Chen L., Su Y., Zhang D., Leng Z., Qi Y., Jiang K. Research on path planning for mobile robots based on improved ACO. Proceedings of the 2021 36th Youth Academic Annual Conference of Chinese Association of Automation (YAC).

[5] Cui Y., Hu W., Rahmani A. (2022). A reinforcement learning based artificial bee colony algorithm with application in robot path planning. Expert Syst. Appl..

[6] Song B., Wang Z., Zou L. (2021). An improved PSO algorithm for smooth path planning of mobile robots using continuous high-degree Bezier curve. Appl. Soft Comput..

[7] Zhou Y., Huang N. (2022). Airport AGV path optimization model based on ant colony algorithm to optimize Dijkstra algorithm in urban systems. Sustain. Comput. Inform. Syst..

[8] Guo S., Pan X., Liu Z. AGV Path Planning Algorithm Based on Fusion of Improved A* and DWA. Proceedings of the 2024 43rd Chinese Control Conference (CCC).

[9] Wu J., Ma X., Peng T., Wang H. (2021). An improved timed elastic band (TEB) algorithm of autonomous ground vehicle (AGV) in complex environment. Sensors.

[10] Zhao H., Song X., Tian Q., Zhang M., Wu D., Han Q. Research on Path Planning of Puncture Robot Based on Improved Artificial Potential Field Method. Proceedings of the 2024 11th International Forum on Electrical Engineering and Automation (IFEEA).

[11] Ravankar A., Kobayashi Y., Hoshino Y., Peng C.-C. (2018). Path Smoothing Techniques in Robot Navigation: State-of-the-Art, Current and Future Challenges. Sensors.

[12] Zhang D., Wang Y., Meng L., Yan J., Qin C. (2024). Adaptive critic design for safety-optimal FTC of unknown nonlinear systems with asymmetric constrained-input. ISA Trans..

[13] Qin C., Qiao X., Wang J., Zhang D., Hou Y., Hu S. (2024). Barrier-Critic Adaptive Robust Control of Nonzero-Sum Differential Games for Uncertain Nonlinear Systems With State Constraints. IEEE Trans. Syst. Man Cybern. Syst..

[14] Zhang D., Hao X., Wang D., Qin C., Zhao B., Liang L., Liu W. (2023). An Efficient Lightweight Convolutional Neural Network for Industrial Surface Defect Detection. Artif. Intell. Rev..

[15] Zhang D., Hao X., Liang L., Liu W., Qin C. (2022). A Novel Deep Convolutional Neural Network Algorithm for Surface Defect Detection. J. Comput. Des. Eng..

[16] Jyotish, Chen M.-Y. A TD-RRT* Based Real-Time Path Planning of a Nonholonomic Mobile Robot and Path Smoothening Technique Using Catmull-Rom Interpolation. Proceedings of the 2022 International Conference on System Science and Engineering (ICSSE).

[17] Ye C., Li J., Jiang H., Zhao H., Ma L., Chapman M. (2020). Semi-automated generation of road transition lines using mobile laser scanning data. IEEE Trans. Intell. Transp. Syst..

[18] Váňa P., Neto A.A., Faigl J., Macharet D.G. Minimal 3D Dubins path with bounded curvature and pitch angle. Proceedings of the 2020 IEEE International Conference on Robotics and Automation (ICRA).

[19] Liu J., Dong X., Wang J., Lu C., Zhao X., Wang X. (2021). A novel EPT autonomous motion control framework for an off-axle hitching tractor-trailer system with drawbar. IEEE Trans. Intell. Veh..

[20] Long C., Shang L., Gao H. Path Smoothing for USVs Using Adaptive Quadratic Bézier Curves. Proceedings of the 2024 4th International Conference on Computer Science, Electronic Information Engineering and Intelligent Control Technology (CEI).

[21] Vailland G., Gouranton V., Babel M. Cubic Bézier local path planner for non-holonomic feasible and comfortable path generation. Proceedings of the 2021 IEEE International Conference on Robotics and Automation (ICRA).

[22] Bae I., Kim J.H., Moon J., Kim S. Lane change maneuver based on Bézier curve providing comfort experience for autonomous vehicle users. Proceedings of the 2019 IEEE Intelligent Transportation Systems Conference (ITSC).

[23] You C., Lu J., Filev D., Tsiotras P. (2020). Autonomous planning and control for intelligent vehicles in traffic. IEEE Trans. Intell. Transp. Syst..

[24] Zhou D., Ma Z., Sun J. (2020). Autonomous vehicles’ turning motion planning for conflict areas at mixed-flow intersections. IEEE Trans. Intell. Veh..

[25] Vorobieva H., Glaser S., Minoiu-Enache N., Mammar S. Automatic parallel parking with geometric continuous-curvature path planning. Proceedings of the 2014 IEEE Intelligent Vehicles Symposium.

[26] Fuji H., Xiang J., Tazaki Y., Levedahl B., Suzuki T. Trajectory planning for automated parking using multi-resolution state roadmap considering non-holonomic constraints. Proceedings of the 2014 IEEE Intelligent Vehicles Symposium.

[27] Ran K., Wang Y., Fang C., Chai Q., Dong X., Liu G. (2024). Improved RRT* Path-Planning Algorithm Based on the Clothoid Curve for a Mobile Robot Under Kinematic Constraints. Sensors.

[28] Liu Y., Liu L., Yu X., Wang C. Optimal Path Planning Algorithm of AUV State Space Sampling Based on Improved Cost Function. Proceedings of the 2020 39th Chinese Control Conference (CCC).

[29] Fan Y., Li L., Zhang F. Intelligent Vehicle Lane-Change Trajectory Planning on Slippery Road Surface Using Nonlinear Programming. Proceedings of the 2022 41st Chinese Control Conference (CCC).

[30] Pandey K.K., Parhi D.R. (2020). Trajectory Planning and the Target Search by the Mobile Robot in an Environment Using a Behavior-Based Neural Network Approach. Robotica.

[31] Zhu D.D., Sun J.Q. (2021). A New Algorithm Based on Dijkstra for Vehicle Path Planning Considering Intersection. IEEE Access.

[32] Zhang T., Wang J., Meng Q. (2022). Generative Adversarial Network Based Heuristics for Sampling-Based Path Planning. IEEE/CAA J. Autom. Sin..

[33] Hu J., Cheng C., Wang C., Zhao C., Pan Q., Liu Z. An Improved Artificial Potential Field Method Based on DWA and Path Optimization. Proceedings of the 2019 IEEE International Conference on Unmanned Systems (ICUS).

[34] Huang Y., Ding H., Zhang Y., Wang H., Gao D., Xu N. (2020). A Motion Planning and Tracking Framework for Autonomous Vehicles Based on Artificial Potential Field Elaborated Resistance Network Approach. IEEE Trans. Ind. Electron..

[35] Deolasee S., Lin Q., Li J., Dolan J.M. Spatio-temporal Motion Planning for Autonomous Vehicles with Trapezoidal Prism Corridors and Bézier Curves. Proceedings of the 2023 American Control Conference (ACC).

[36] Wulfmeier M., Rao D., Wang D.Z., Ondruska P., Posner I. (2017). Large-scale Cost Function Learning for Path Planning Using Deep Inverse Reinforcement Learning. Int. J. Robot. Res..

[37] Cuesta R., Alvarez J. Limit Cycle Construction Using Bézier Curves: Application in a Mobile Robot. Proceedings of the 2023 XXV Robotics Mexican Congress (COMRob).

[38] Li X., Sun Z., Kurt A., Zhu Q. A Sampling-Based Local Trajectory Planner for Autonomous Driving Along a Reference Path. Proceedings of the 2014 IEEE Intelligent Vehicles Symposium Proceedings.

[39] Guo M., Peng J., Huang H. (2023). Unmanned Vehicle Path Planning and Tracking Control Based on Improved Artificial Potential Field Method. J. Syst. Simul..

[40] Zhou H., Meng T., Sun S. Asymptotically Time-Optimal Smooth Trajectory Planning in Dynamic Environments. Proceedings of the 2024 China Automation Congress (CAC).

[41] Cai W., Gan Y., Fang F., Zhou B., Dai X. Smooth and Time-optimal Improved-Bezier Trajectory Planning Based on Dynamics for Industrial Robots. Proceedings of the 2024 43rd Chinese Control Conference (CCC).

[42] Wang X., Gan Y., Dai X. Time-Optimal and Smooth Trajectory Planning Based On Continuous Pseudo-Acceleration. Proceedings of the 2023 International Conference on Advanced Robotics and Mechatronics (ICARM).

[43] Zhao H., Liu Y., Cheng W., Jiang Z., Xiao B., Wu Y. Node Control-Bidirectional RRT: Fast and Smooth Trajectory Planning for Live Working Robot. Proceedings of the 2022 IEEE International Conference on Robotics and Biomimetics (ROBIO).

